# A Simplified Approach for the Corrosion Fatigue Assessment of Steel Structures in Aggressive Environments

**DOI:** 10.3390/ma15062210

**Published:** 2022-03-17

**Authors:** Aldo Milone, Raffaele Landolfo

**Affiliations:** Department of Structures for Engineering and Architecture, University of Naples “Federico II”, Via Forno Vecchio 36, 80134 Naples, Italy; aldo.milone@unina.it

**Keywords:** steel structures, corrosion, fatigue assessment, material degradation

## Abstract

Fatigue performance is often a key aspect when dealing with existing steel structures such as steel bridges or offshore constructions. This issue proves to be more critical as these structures are usually located in aggressive environments and are thus exposed to progressive degradation. Indeed, disruptive phenomena such as corrosion can severely worsen the fatigue performance of the steel components. Currently, the normative standards do not provide a codified procedure for the fatigue checks of steel structures subjected to ongoing corrosion. Within this framework, in this paper a simplified approach for the life-cycle assessment of corroded steel structures is proposed. For this purpose, the concept of “critical corrosion degree” is introduced, allowing the expression of corrosion fatigue checks in a more direct “demand vs. capacity” form with respect to the currently available methods. A first validation of such methodology is reported for the corrosion fatigue tests drawn from the literature. The predicted levels of critical corrosion are in good agreement with the values of artificially induced corrosion (i.e., 4, 8, and 12% of mass loss, respectively), with a maximum relative error of ≈9.3% for the most corroded specimen. Finally, parametrical analyses are performed, highlighting the influence of the model parameters on the corrosion fatigue performance of the steel elements.

## 1. Introduction

The fatigue performance of steel structures has become a relevant topic for civil engineering since the second half of the 20th century, due to the occurrence of some relevant fatigue-related failures [1,2]. Several efforts to understand fatigue phenomenology have been made since, leading to the publication of normative requirements for fatigue verifications of steel structures worldwide. With regard to the European countries, such provisions are currently codified in EN1993:1–9 [3], which provides two alternative philosophies for the fatigue checking of steel structures, namely:

- The Safe-Life (SL) approach, which does not contemplate any fatigue damage in checked structures. The SL approach is addressed by means of a punctual stress-based verification, i.e., it refers to the worst load conditions occurring in the service life;
- The Damage-Tolerant (DT) approach, which admits the development of controlled fatigue damage in verified structures. Expected damage, which is estimated accounting for the entire service life, should not exceed a threshold value associated with failure.

When dealing with existing steel structures, the DT approach is clearly the most suitable option as it allows for the accounting of the damage endured by steel elements during their past service life. The amount of cumulated damage can be often significant for older structures, mainly owing to the inadequacy of the design requirements available at the erection time and/or to a progressive increase in cyclic loads over the years.

Moreover, in the case of existing steel structures a further relevant source of damage is represented by material degradation, mainly in the form of metallic corrosion [4].

Corrosion consists of progressive material loss due to an electrochemical process which is extremely sensitive to local environmental conditions (i.e., temperature, relative humidity, salinity, etc.) [5,6]. In particular, extensive portions of exposed structures can be affected by degradation or, conversely, damage can take place in highly localized spots (non-uniform corrosion) [7,8,9,10]. At the same time, the material loss rate can significantly vary during structural service life in dependence on the above parameters.

When corrosive phenomena occur in combination with cyclic loadings, corrosion-induced damage does not simply add up to fatigue damage, but rather, the two processes influence each other. Multiple factors underlie this mutual interaction, namely [11]:

- On one hand, corrosion induces a localized reduction of the resisting cross-section, resulting in stress amplifications which accelerate fatigue cracking. Moreover, the fatigue strength of iron oxide products is considerably lower in comparison to that of pristine steel;
- On the other hand, fatigue cracking creates preferable spots for corrosion development as the cracks can penetrate through protective layers, if present.

It should be remarked that both phenomena are characterized by a significant degree of randomness [1,2,5,9,10], which further intensifies when fatigue and corrosion damage are combined. To address this aspect, two main approaches have been followed in the scientific literature, namely (i) implementing stochastic procedures to account for both fatigue and corrosion intrinsic randomness and (ii) using simplified techniques to deal with corrosion fatigue in a deterministic way, usually based on experimental data. Nevertheless, it is clear that the phenomenology of corrosion fatigue in steel structures is a complex topic which still represents an open field of research nowadays [12,13,14,15].

Therefore, it is not surprising that no codified procedures for the corrosion fatigue assessment of steel constructions are available at present time. This lack of provisions is indeed crucial as steel structures, which are conceived to endure relevant cyclic loads during their service life, are often placed in aggressive environments (e.g., steel bridges or offshore structures) [16]. Nevertheless, some relevant contributions dealing with the corrosion fatigue analysis of steel structures can already be found in the scientific literature.

With regard to the stochastic approaches, Lehner et al. [17] investigated the effectiveness of Monte Carlo simulations for the fatigue analysis of a riveted crane support truss that had been in service for nearly 100 years, in order to account for the random nature of cyclic loads.

Yanez-Borjaz et al. [18] took advantage of transient load randomness to detect corrosion fatigue damage in a truss bridge structure. Indeed, the authors succeeded in identifying three sequential damage levels based on the dynamic structural response under random vibrational signals.

Li et al. [19] modelled corrosion fatigue as a stochastic process in order to assess the time-dependent failure probability of corroded riveted connections employed in a steel bridge. The results highlighted that corrosion greatly increases failure probability at a given time with respect to lone fatigue damage.

Moreover, the authors proposed a modification of S-N curves (i.e., increasing the logarithmic slope of Wohler curves proportionally to the corrosion degree *η*) to account for this effect for both high-cycle (HCF) and low-cycle fatigue (LCF).

Within the framework of simplified fatigue assessment procedures, Landolfo et al. [20] investigated the fatigue performance of an existing riveted railway bridge affected by corrosion, considering the effect of progressive thickness reduction on the fatigue demand, although no reduction in fatigue strength due to corrosion was accounted for.

This effect has indeed been experimentally investigated by several authors. For instance, Adasooriya et al. [21] demonstrated that steel components in aggressive environments show a significant decline of fatigue performance, which can be expressed by modifying the slopes of the reference S-N curves. According to the authors, the logarithmic slopes of both the LCF and the HCF branches of the S-N curves can reach up to ≈1.6 times their original values in the worst conditions, i.e., in the case of exposure to saline water.

Similar outcomes have been found by Jiang et al. [22] with respect to details adopted in stranded bridges. Namely, the authors proposed a corrosion fatigue domain in which the shape of the S-N curves depends on the corrosion degree *η*, such as with the logarithmic slope linearly increasing for increasing values of *η*. It is worth observing that the findings reported in [21,22] both comply with the results derived by Li et al. [19].

Wu et al. [23] confirmed how neglecting the effect of corrosion can lead to a significant underestimation of fatigue damage. The authors investigated a large-span suspended bridge with a truss deck as a case study, showing how the exposure to an aggressive environment can induce a reduction of ≈30% in terms of expected service life with respect to lone fatigue calculations.

Within the framework of deterministic assessment techniques, a simplified approach for the prediction of the fatigue performance of corroded steel elements is presented in this paper. This work aims at reformulating fatigue checks in an explicit “demand vs. capacity” form, which is more in line with the principles of performance-based engineering. For this purpose, the concept of ”critical corrosion degree” η*_Rd_, associated with an assigned target fatigue life t*, is introduced.

The present paper is mainly divided into three parts. In the first part, the proposed procedure is presented and discussed in detail. In the second part, a first validation of the presented methodology is reported with reference to the corrosion high-cycle + low-cycle fatigue (HCF+LCF) experimental trials performed by Sun et al. [24]. Finally, parametrical analyses based on the same fatigue tests are performed in order to highlight the influence of the governing parameters on the corrosion HCF+LCF performance of the structural details.

## 2. Corrosion Fatigue Assessment of Steel Structures: Methods

The proposed method for the corrosion fatigue assessment of steel structures in aggressive environments is based on the well-known Palmgren–Miner’s rule [25] for damage cumulation, which is already codified in EN1993:1–9 within the framework of the DT approach [3]. Nevertheless, in this work, fatigue checks will be presented in a more direct “capacity vs. demand” approach within the framework of performance-based engineering. This aspect is addressed by introducing the concept of “critical corrosion degree” η*_Rd_, which is derived with respect to an assumed target fatigue life t*.

**Figure 1:**
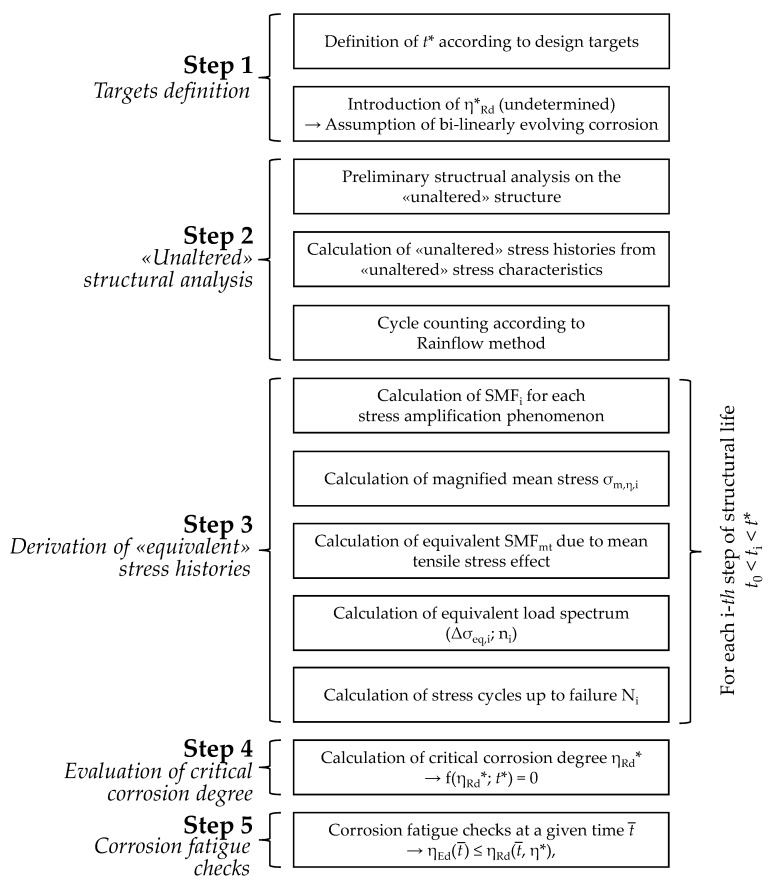
Articulation of the proposed methodology for the fatigue assessment of steel structures in aggressive environments.

The main stages of the presented procedure can be summarized as follows (see Figure 1):

- Step 1: Definition of target fatigue life t* and selection of the corrosion trend according to exposition conditions (see Section 2.1);
- Step 2: “Unaltered” structural analysis on the pristine structure, i.e., provisionally disregarding the effects of corrosion (see Section 2.2);
- Step 3: Derivation of “equivalent” load spectrums accounting for corrosion-induced local stress amplifications (see Section 2.3);
- Step 4: Estimation of critical corrosion degree η*_Rd_ by means of a target function *f*(*η*, t*) (see Section 2.4);
- Step 5: Corrosion fatigue checks in “demand/capacity” form (see Section 2.5).

Each of the introduced steps will be discussed in detail in the following subsections.

### 2.1. Step 1: Definition of Target Design Life and of Corrosion Development

As highlighted in the introduction section, the corrosive processes are extremely sensitive to local boundary conditions. In general, in the years immediately after the construction time, corrosion is limited due to the presence of preventive measures. Once the protection layer is completely worn, the corrosion process sharply accelerates.

**Figure 2:**
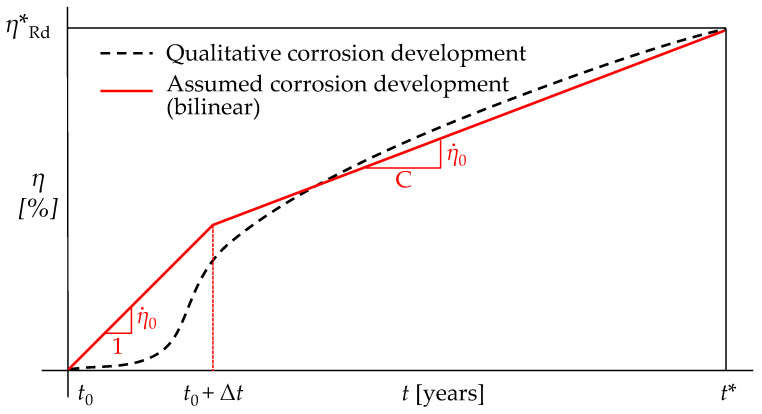
Qualitative (black dashed curve) and assumed (red solid polyline) trends for corrosion development for the proposed methodological approach.

Therefore, after significant quantities of superficial corrosion products have already formed, the corrosive process generally slows down, approaching a stabilized rate of progression (See Figure 2, dashed black curve—qualitative corrosion development) [4,9,26]. Several attempts have been made in the scientific literature to analytically describe this trend, i.e., by means of broken lines or more sophisticated power laws, which appeared particularly suitable in the case of highly localized corrosion [7,8,26].

Nevertheless, it should be remarked that the instantaneous corrosion rate $\dot{\eta }$ (i.e., the time derivative of the corrosion degree d*η*/d*t* at a given time) varies sensibly through the structural service life in dependence on multiple factors (temperature, relative humidity, aggressive agent concentration, etc.). Thus, predicting the actual trend of corrosion degree against time is a complex task without the aid of a constant structural monitoring.

Moreover, even when the necessary information is available, the analysis of corrosion development is highly impractical and not free from a consistent degree of uncertainty.

Therefore, according to prescriptions from UNI EN ISO 9224 [27], in the presented approach the corrosion development is approximated by a polyline with two branches (i.e., with a piecewise constant corrosion rate; see Figure 2, red solid line—assumed corrosion development).

The ratio among the slopes of the consecutive branches is equal to C: 1, with C ≥ 1 depending on the corrosivity category of concern. The suggested values of parameter C for each category are summarized in Table 1.

The knee point of the bi-linear curve is assumed to occur Δ*t* years after construction time *t*_0_. According to the recommendations from [27], in the absence of more precise information, Δ*t* = 10 years should be selected.

The corrosion degree *η* is assumed null for *t* = *t*_0_. Conversely, in correspondence with the target fatigue life t*, a “critical corrosion degree” η*_Rd_ is introduced, i.e., the minimum corrosion degree which exactly induces corrosion fatigue collapse for the assumed t*.

It should be remarked that this assumption, i.e., monotonically increasing *η*(*t*), can be considered sufficiently accurate in the case of scarcely maintained structures, while it will lead to conservative estimations in the case of properly preserved structures as protective measures such as coatings and polishes can temporarily arrest corrosion development [4,10].

The selection of a proper value for t* has to be intended as a designer’s choice, which depends on the expected influence of both the fatigue and the material degradation on structural performance. Nevertheless, owing to the sudden and undesirable nature of corrosion fatigue failures, t* should always be sufficiently greater than the design’s working life *L_wd_*. For this purpose, an appropriate fatigue life multiplier *k* > 1 can be introduced, i.e., the ratio of t* and *L_wd_*.

Hence, in the absence of more detailed information, for structures in which fatigue already governs the design choices, smaller values of *k* can be assumed (that is, *k* = 1.1 ÷ 1.2). Conversely, for structures in which fatigue collapse is not expected and/or for which a significant ultimate ductility is required, higher values of *k* should be selected (that is, *k* = 1.5 ÷ 2.0). Nevertheless, it should be remarked that more accurate values of *k* can be derived on a case-by-case basis according to reliability considerations, in compliance with the EN1990 previsions [28].

Consistently with the assumption of a piecewise constant corrosion rate, the corrosion degree at a given time *η*(*t*) within the service life is expressed by Equation (1):

(1) $$\eta \left(t\right)=\{\begin{array}{cc} {\dot{\eta }}_{0}\left(t-t_{0}\right) & t\leq t_{0}+\Delta t\\ \frac{{\dot{\eta }}_{0}}{C}\left(t-t_{0}\right)+\Delta t{\dot{\eta }}_{0}\left(1-\frac{1}{C}\right) & t_{0}+\Delta t<t\leq t^{*} \end{array}$$

(2) $$\dot{\eta_{0}}=\frac{\eta_{Rd}^{*}}{\Delta t\left(1-\frac{1}{C}\right)+\frac{t^{*}}{C}}$$

in which $\dot{\eta_{0}}$ represents the corrosion rate assumed for the first branch of the polyline, which, for a given corrosivity category, is only a function of η*_Rd_ and t* (see Equation (2)). Equations (1) and (2) hold true for an arbitrary value of η*_Rd_, which is still undetermined in Step 1. The estimation of the actual value of η*_Rd_ will be addressed in Step 4 (see Section 2.4 for further details).

### 2.2. Step 2: “Unaltered” Structural Analysis

A common assumption made in the global analysis of corroded structural members is to neglect any self-weight and stiffness reduction due to mass loss [10,12]. This assumption can be considered sufficiently accurate since, according to [27], the expected values of material loss do not exceed a few mg/(year·m^2^), even in the case of extremely aggressive environments (i.e., for corrosivity category C5). Moreover, in most circumstances neglecting stiffness degradation leads to a conservative estimation of the stress characteristics in corroded structural members. Indeed, while no stress redistribution is expected for isostatic damaged structures, in the case of redundant structures corrosion would result in stress migration towards the intact elements.

Therefore, structural analysis may be performed considering the “unaltered” structure in order to evaluate the values of the stress characteristics (*S*_0_) only once. The influence of corrosion in terms of local stress amplifications will be considered for a second time by means of the appropriate magnification factors.

The results of “unaltered” structural analysis should account separately for the permanent and transient load as in most cases the former single-handedly contributes to the definition of minimum stresses in the structural elements (*S*_0,min_), while the latter induces stress fluctuations (Δ*S*_0_) influencing the maximum values of the stress characteristics (*S*_0,max_).

### 2.3. Step 3: Evaluation of “Equivalent” Corrosion-Depending Load Spectrums

Once the “unaltered” stress characteristics *S*_0,min_/Δ*S*_0_ have been determined, the “unaltered” local stresses can be determined using the well-known expressions from the theory of elasticity. Therefore, for each time during the service life, the “real” corrosion-depending stresses σ_η,min_/Δσ_η_ can be determined by amplifying σ_0,min_/Δσ_0_ by the means of stress magnification factors (SMFs).

In general, when dealing with steel structures in aggressive environments, at least two sources of stress amplification should be considered, i.e., the local reduction of the resisting cross-section induced by corrosion and the presence of mean tensile stresses σ_0,m_ > 0 under pulsating loads [29]. More properly, the latter condition results in a reduction in fatigue strength rather than an increase in fatigue demand, although several studies have highlighted that the mean stress effect can be accounted for by amplifying the design stress range up to an equivalent value [30,31,32].

Corrosion-induced amplification can be modelled by means of an SMF (henceforth referred to as *SMF_η_*), which is a function of (i) the pristine resisting cross-section properties *X*_0_ (in which *X* can stand for area, second moment of area, etc., depending on the monitored stress characteristics); (ii) the cross-section properties reduction Δ*X_η_*; and, generally speaking, (iii) the nature of the corrosive process (*CP*), as follows (Equation (3)):

(3) $$SMF_{\eta }=f\left(X_{0},\Delta X_{\eta },\;CP,\;\eta_{Rd}^{*},t\right)\geq \;1$$

Indeed, as corrosive processes depend on local boundary conditions, corrosion can induce strongly localized damage in small portions of the structural members in the form of sharp “pits” [5,9]. This phenomenon, which is known as “pitting corrosion”, can induce local stress magnifications which overly exceed the values estimated for uniform corrosion, i.e., the condition in which material loss is homogenously spread along the element. Uniform corrosion represents the most favorable condition for damaged steel structures as stress amplifications attain a minimum value, which can be expressed as follows (Equation (4)):

(4) $$SMF_{\eta ,uniform}=\frac{1}{1-\frac{\Delta X_{\eta }}{X_{0}}}=\underset{CP}{\min}\left(SMN_{\eta }\right)$$

“Real” corrosion-depending stresses *σ_η_*_,min_/Δ*σ_η_* are hence derived as follows (Equations (5) and (6)):

(5) $$\sigma_{\eta ,\text{min}}=SMF_{\eta }\;\sigma_{0,\text{min}}$$

(6) $$\Delta \sigma_{\eta }=SMF_{\eta }\;\Delta \sigma_{0}$$

As in most cases “unaltered” stress histories due to transient loads are already complex and aperiodic, it is clear that “real” stress histories cannot be described by a single value Δ*σ_η_*, but should, rather, be expressed in the form of “real” oscillograms, in which each point *σ_η_*(*t*) is derived by the amplifying of *SMF_η_* times the related “unaltered” stress *σ*_0_(*t*).

At this point, cycle counting is performed considering only the fluctuating part of the amplified stresses Δ*σ_η_*. For this purpose, both the Reservoir and the Rainflow methods (which are codified in EN:1993-1-9 [3]) can be used, although the latter procedure is more suitable in light of a computational implementation of the presented methodological approach [2].

Cycle counting yields a first “approximation” of the corrosion-depending load spectrum (Δ*σ_η,i_*; *n_i_*). The equivalent fatigue demand Δ*σ_eq,i_* is finally estimated considering the effect of the mean tensile stresses, as follows (Equation (7)):

(7) $$\Delta \sigma_{eq,i}\left(t\right)=\Delta \sigma_{\eta ,i}\left(t\right)\cdot SMF_{Eq}\left(t\right)$$

where *SMF_Eq_* is an equivalent magnification factor accounting for the mean stress effect. According to the experimental evidence and consolidated practice in fatigue analysis [2], Goodman’s model [30] is selected in this work to deal with pulsating stress histories.

It should be remarked that *SMF_Eq_* implicitly depends on Δ*σ_η,i_*, as Equations (8) and (9) hold true:

(8) $$SMF_{Eq}\left(t\right)=\frac{1}{1-\frac{\sigma_{\eta ,m,i}\left(t\right)}{f_{u}}}$$

(9) $$\sigma_{m,\eta ,i}\left(t\right)=\sigma_{\eta ,min,i}\left(t\right)+\frac{\Delta \sigma_{\eta ,i}\left(t\right)}{2}$$

with *f_u_* being the ultimate tensile strength (UTS) of structural steel.

As shown by González-Arévalo et al. [33], steel properties can actually deteriorate in time due to material aging, resulting in accelerated damage evolution. Specifically, up to a 4% decrease in UTS can be found in aged steels with respect to the original value. Therefore, in the case of structures conceived to endure for a very long service life, this effect can be accounted for by introducing a time dependence for *f_u_*.

**Figure 3:**
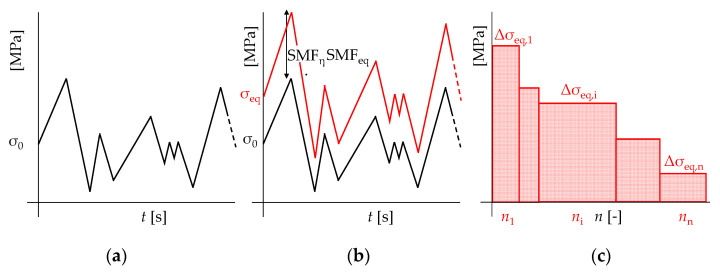
Estimation of “real” load spectrum from aperiodic stress histories according to the proposed methodological approach: (**a**) “unaltered” stress oscillograms, (**b**) “real” and “equivalent” stress oscillograms, and (**c**) “equivalent” load spectrums.

In this way, accelerated damaging due to steel aging can be equivalently considered, i.e., with a further increase of *SMF_Eq_*(*t*) in comparison to the value deriving from the lone mean stress effect. The evaluation of Δ*σ_eq,i_* is performed for each stage of the structural service life, thus obtaining (t*–*t*_0_) “equivalent” load spectrums, which represent the overall fatigue demand on the analyzed steel structure in an aggressive environment (Figure 3).

### 2.4. Step 4: Evaluation of Critical Corrosion Degree η*_Rd_

Using Palmgren–Miner’s rule for linear damage cumulation, the actual value of the critical corrosion degree η*_Rd_ can be estimated by imposing that the total damage parameter *D_TOT_* reaches unity for *t* = t*. This operation can be performed numerically by solving the following implicit equation for η*_Rd_ (Equation (10)):

(10) $$f\left(\eta_{Rd}^{*},t^{*}\right)=1-D_{TOT}\left(\eta_{Rd}^{*},t^{*}\right)=1-\overset{t^{*}}{\underset{t=t_{0}}{\sum }}\frac{n_{i}}{N_{i}\left(\Delta \sigma_{eq,i}\left(\eta_{Rd}^{*}\right)\right)}-1=0$$

It is worth reporting that Equation (10) assumes the damage threshold associated with fatigue collapse (D_TOT_*) as a fixed quantity a priori, i.e., D_TOT_* = 1.

This simplification, although widely accepted in both the literature and design practice [2], does not allow accounting for the intrinsically random nature of the fatigue phenomenon. Indeed, as highlighted by Aid et al. [34], the randomness exhibited by both the material properties and the loading histories mainly contributes to fatigue stochasticity.

In the spirit of preserving the simplified nature of the presented procedure, this aspect can be addressed by considering a different value for D_TOT_* while solving Equation (10).

As reported by [19], D_TOT_* approximately behaves as a log-normal random variable with expected value E(D_TOT_*) = 1 and COV = 0.3. Therefore, η*_Rd_ can be computed within a certain confidence interval by considering the appropriate values of D_TOT_* ≠ 1.

Nevertheless, it should be remarked that Equation (10) still yields a reliable estimation of the critical corrosion degree due to η*_Rd_ being associated with the expected value of D_TOT_* = 1.

**Figure 4:**
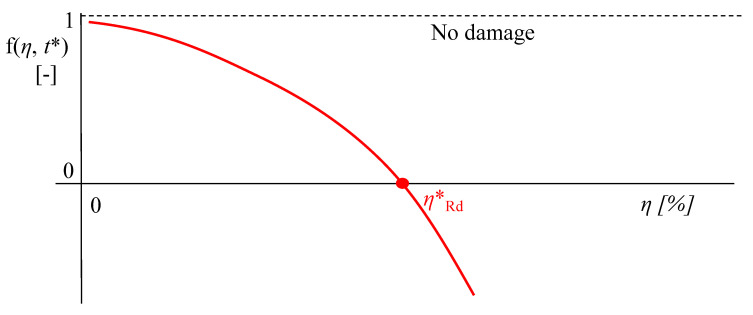
Monotonic trend of *f*(*η*, t*) and corresponding value of η*_Rd_ for *f*(*η*, t*) = 0.

The numerical solution of Equation (10) only requires a few iterations as D_TOT_ monotonically decreases for increasing values of η*_Rd_ (See Figure 4).

In order to account for fatigue strength reduction due to material degradation, the evaluation of the number of stress cycles up to failure *N*_i_ has to be performed using a S-N-*η* fatigue strength domain in place of the S-N curves provided by EN1993:1–9 [3]. On the basis of the results reported in [19,21,35], the following expressions can be used (Equations (11) and (12)):

(11) $$\log\frac{5\times 10^{6}}{N_{i}}=m_{1,\eta }\log\left(\frac{\Delta \sigma_{D,\eta }}{\Delta \sigma_{Eq,i}}\right)$$

(12) $$\log\frac{N_{i}}{5\times 10^{6}}=m_{2,\eta }\log\left(\frac{\Delta \sigma_{Eq,i}}{\Delta \sigma_{D,\eta }}\right)$$

with Δ*σ_D,η_* being the modified constant amplitude fatigue limit (CAFL) for the selected structural member, accounting for the effect of corrosion (i.e., the stress range which induces corrosion fatigue failure for *N* = 5 × 10^6^), and *m*_1,*η*_/*m*_2,*η*_ being the corrosion-depending inverse slopes of the LCF and HCF branch of the S-N curve, respectively.

According to [21,35], the modified CAFL can be derived assuming that very low-cycle fatigue (VLCF) behavior is unaffected by corrosion, i.e., the stress range Δ*σ*_10000_ inducing fatigue collapse for *N* = 10^4^ does not depend on *η*. Thus, Δ*σ_D,η_* can be calculated through Equation (13):

(13) $$\Delta \sigma_{D,\eta }=\frac{\Delta \sigma_{10000}}{{\left(5\times 10^{2}\right)}^{\frac{1}{m_{1,\eta }}}}$$

Δ*σ*_10000_ is immediately derived from the detail class for the structural member Δ*σ_C_*, i.e., the stress range causing fatigue collapse for *N* = 2 × 10^6^ in the absence of corrosion (Equation (14)):

(14) $$\Delta \sigma_{D,10000}={\left(2\times 10^{2}\right)}^{\frac{1}{m_{1,0}}}\cdot \Delta \sigma_{C}$$

**Figure 5:**
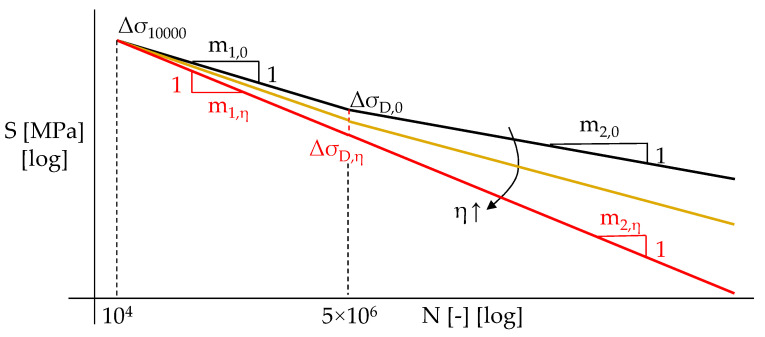
Assumed shape of S-M-*η* domains for corrosion fatigue analyses.

For the sake of clarity, *m*_1,0_ is the inverse slope of the LCF branch according to EN1993:1–9 [3] in pristine conditions. Consistently with the results reported in [19,22], the inverse slopes are assumed to linearly reduce as *η* increases, as follows (Equations (15) and (16)):

(15) $$m_{1,\eta }=m_{1,0}-\frac{\eta }{\eta_{ref}}\Delta m_{1,\eta }$$

(16) $$m_{2,\eta }=m_{2,0}-\frac{\eta }{\eta_{ref}}\Delta m_{2,\eta }$$

in which *m*_1,0_/*m*_2,0_ are the inverse slopes of the S-N curve for LCF/HCF in pristine conditions, respectively; *m*_1,*η*_/*m*_2,*η*_ are the corrosion-affected inverse slopes for the same branches; and *η_ref_*/Δ*m*_1,η_/Δ*m*_2,η_ are the experimental parameters expressing the influence of corrosion on the fatigue behavior of the steel components. According to the results from [21,35], *η_ref_* = 0.2, Δ*m*_1,*η*_ = 0.375 *m*_1,0_, and Δ*m*_2,*η*_ = 0.375 *m*_2,0_ are, hence, assumed. In this way, the observed increase of the slopes (i.e., ≈1.6 times their original values) is attained for the mean value of corrosion detected in the reference experimental tests (i.e., *η_mean_* ≈20%). Conservatively, and in line with the experimental evidence [10,29,36], for the corrosion fatigue domain no endurance limit is assumed, i.e., *m*_2,_*_η_* < ∞. The resulting shape of the S-N-*η* domains, according to Equations (11)–(16), is depicted in Figure 5 for the different levels of *η*.

### 2.5. Step 5: Corrosion Fatigue Checks

Once the value of η*_Rd_ is known, the corrosion fatigue checks are immediately performed by controlling that “corrosion demand” at a given time *η_Ed_* ($\bar{t}$) does not exceed the “corrosion capacity” *η_Rd_* ($\bar{t}$) for the same $\bar{t}$, which is simply derived from η*_Rd_ according to the assumption of the piecewise constant corrosion rate (Equation (17)):

(17) $$\eta_{Ed}\left(\bar{t}\right)\;\leq \eta_{Rd}\left(\bar{t}\right)=\eta_{Rd}^{*}-\frac{\eta_{Rd}^{*}}{\Delta t\left(C-1\right)+t^{*}}\left(t^{*}-\bar{t}\right)$$

The main strength of the presented approach lies in the fact that η*_Rd_ is a constant reference value which can be easily determined numerically, thus allowing the calculation *η_Rd_*($\bar{t}$) for a generic instant of assessment $\bar{t}$, while *η_Ed_*($\bar{t}$) can be directly estimated on the basis of on-field monitoring evidence. On the other hand, significant uncertainties regarding load histories (for example, in the case of wind-induced fatigue in aggressive environments) or corrosion trends which sensibly differ from the assumed expression for *η*(*t*) (see Equation (1) for example, in the case of periodically maintained structures) may negatively affect the reliability of the presented procedure.

**Figure 6:**
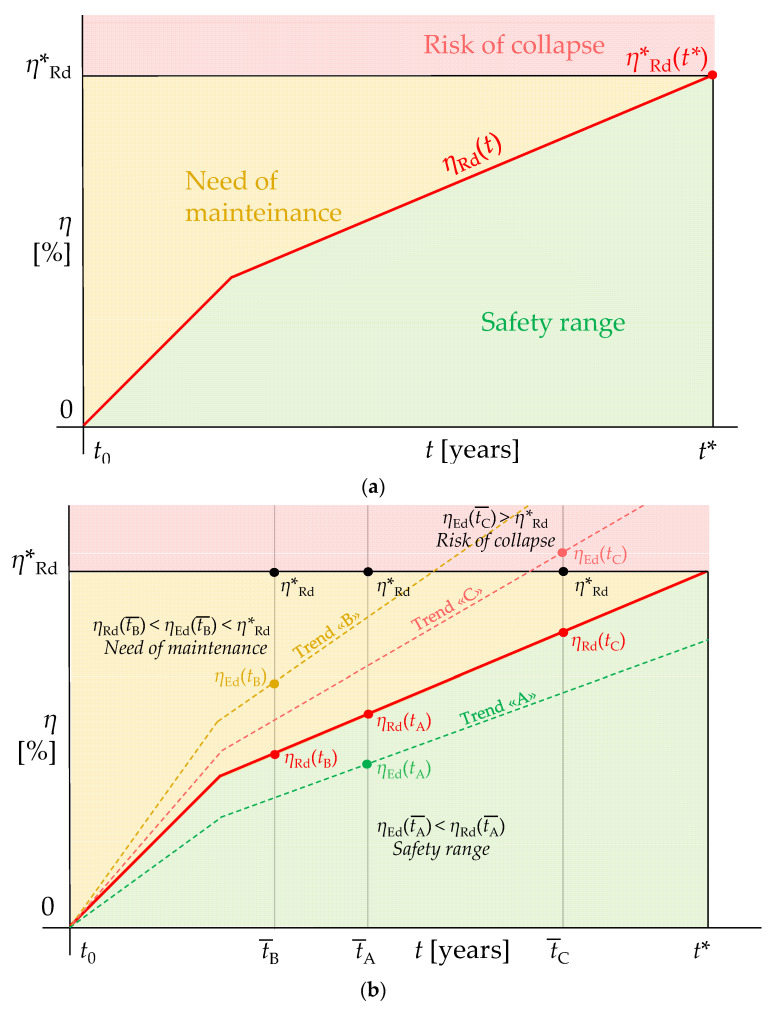
(**a**) Damage stages due to corrosion fatigue according to the presented methodological approach and (**b**) examples of fatigue check results for different corrosion trends and instants of assessment.

On the basis of the results of the corrosion fatigue checks, three different damage stages can be identified for the investigated structure, namely (Figure 6a):

- For 0 < *η_Ed_* (*t*) < *η_Rd_* (*t*), the structure can be considered safe with regard to corrosion fatigue failure. Nevertheless, periodic inspections should be carried out to verify if this condition holds true in time (Figure 6b, Trend “A”);
- For *η_Rd_* (*t*) < *η_Ed_* (*t*) < η*_Rd_, the necessity of maintenance measures emerges as values of η > η*_Rd_ are expected to be attained prior to the target fatigue life t* (Figure 6b, Trend “B”);
- For *η_Ed_* (*t*) > η*, the structure quickly requires safety measures as risk of collapse due to corrosion fatigue is predicted (Figure 6b, Trend “C”).

## 3. Validation against Corrosion Fatigue Tests Drawn from Literature

In the previous Section, each stage of the proposed methodological approach was introduced and widely discussed. Hence, a first validation for the described procedure is presented with regard to the elasto-plastic corrosion fatigue tests drawn from the work of Sun et al. [24].

Corrosion HCF+LCF trials are hence selected to emulate realistic conditions for existing steel structures, such as road and railway bridges, in which structural deficiencies (often found due to the inadequacy of normative provisions in force at erection time) can induce unforeseen local plasticization in highly stressed members and also in light of the progressive increase in traffic loads up to present time [37]. Such conditions may result in significant damage accumulation, which adds up with the ongoing material degradation and often remains undetected if no structural monitoring is provided.

In this case, the subsequent effect of exceptional traffic loads or even moderate earthquakes can induce premature failure due to LCF in an aggressive environment.

Sun et al. performed HCF+LCF corrosion fatigue trials on coupon tests subjected to artificial corrosion in order to highlight the influence of corrosive processes on the fatigue performance of AH32 steel, which is widely used in bridge and marine engineering due to its relatively high tensile strength and good corrosion resistance.

AH32 steel has a nominal yielding strength *f_y_* = 315 N/mm^2^, while its tensile strength *f_u_* ranges within 440 ÷ 590 N/mm^2^.

Corrosion was artificially induced on the tested specimen using a sodium chloride solution (3.5%) as a corrosive medium. Direct current having a density equal to 1000 μA/cm^2^ was hence applied using a copper element as a conductor. The duration of the corrosive process was set according to Faraday’s law to reach the target values of material loss (i.e., 4, 8, and 12% for specimens *S2*-*S3*-*S4*, respectively). Epoxy resin was applied on the coupon ends to avoid occurrence of corrosion outside the gauge length. The corrosion fatigue behavior of the specimen was therefore investigated by means of HCF+LCF stress-controlled protocols. Namely, 10,000 pulsating cycles having a nominal stress range of Δ*σ_0_* < *f*_y_ were applied before exceeding yielding strength for the remaining cycles up to failure.

The fatigue behavior of a pristine specimen (henceforth referred to as “*S1*”) was assumed as a benchmark to investigate the actual effect of corrosion on the cyclic behavior of AH32 steel. Three different degrees of corrosion were considered for experimental tests, namely *η* = 4% (Specimen “*S2*”), *η* = 8% (Specimen “*S3*”) and *η* = 12% (Specimen “*S4*”).

The test conditions for specimens *S1–S4* are summarized in Table 2.

One can notice that both the number of cycles *n*_1_ and the mean stress *σ_m,_*_1_ were kept constant for each specimen during the elastic loading block, while a stress ratio of *R*_2_ = 0.1 was adopted for all tests during the second elasto-plastic cycles block.

With regard to the test results reported in [24], the presented methodological approach is adopted to assess whether the estimated critical corrosion degrees η*_Rd__,S2-to-S4_ are comparable with the maximum corrosion degrees η*_max,S2-to-S4_ artificially induced on the tested specimens.

Moreover, in order to avoid possible errors induced by the excessive conservativity of the design S-N curves provided by [3], the results for the pristine specimen (*S1*) are priorly used to calibrate an “equivalent” detail class Δ*σ_C,AH32_* for the AH32 steel coupon tests.

As the proposed procedure is applied to perform post-failure analyses, the target fatigue life t* for each test is a priori known. All specimens were tested adopting a constant loading frequency (i.e., 5 Hz). Therefore, t* is directly related to the number of cycles up to failure N_i_*. Hence, for the sake of convenience, the outcomes from the proposed methodology will be presented in terms of cycle-depending rather than time-depending results.

According to the test conditions, the coupons were subjected to lone axial stresses, and artificial corrosion was induced only within the gauge segment length *L_g_*.

Therefore, the simplified assumption of uniform corrosion can be considered sufficiently accurate, allowing for approximate corrosion degree *η* in terms of relative cross-section loss *η_A_*, as follows (Equation (18)):

(18) $$\eta \left(t\right)\approx \eta_{A}\left(t\right)=\frac{\Delta A_{\eta }\left(t\right)}{A_{0}}$$

with Δ*A_η_*(t) being the reduction of the resisting cross-section of the gauge segment at a given time and *A*_0_ = 28.3 mm^2^ being the pristine area of the gauge segment.

With respect to the exposition conditions, the artificially induced corrosion has to be clearly regarded as the effect of an extremely aggressive environment. Therefore, *C* = 1 is selected to define the corrosion trend (i.e., constant corrosion rate d*η*/d*t*).

In light of the above, the critical corrosion degree η*_Rd_ is assumed to be approximately equal to the critical relative area loss η_A_*.

“Unaltered” stress oscillograms are a priori known according to test conditions. Hence, “equivalent” load spectrums accounting for both corrosion- and mean-stress-induced amplifications can be directly derived using the proper stress magnification factors. With reference to the selected tests, the SMFs can be expressed as follows (Equations (19) and (20)):

(19) $$SMF_{\eta }\left(t\right)=\frac{1}{1-\eta_{A}\left(t\right)}$$

(20) $$SMF_{Eq}\left(t\right)=\frac{1}{1-\frac{1+R}{2\left(1-R\right)}\frac{\Delta \sigma_{\eta }\left(t\right)}{f_{u}}}$$

Equations (17) and (18) clearly highlight how the two amplifying effects are coupled as *SMF_Eq_* depends on the “real” stress range Δ*σ_η_* = *SMF_η_*·Δ*σ_0_*.

In order to quantify the influence of the mean stress effect, in the absence of more detailed information, *f_u_* = 515 N/mm^2^ was assumed, i.e., the mean value within the range of possible UTS for AH32 steel (440 ÷ 590 N/mm^2^). Nevertheless, the sensitivity of the presented procedure with respect to this assumption will be investigated in the following section.

**Figure 7:**
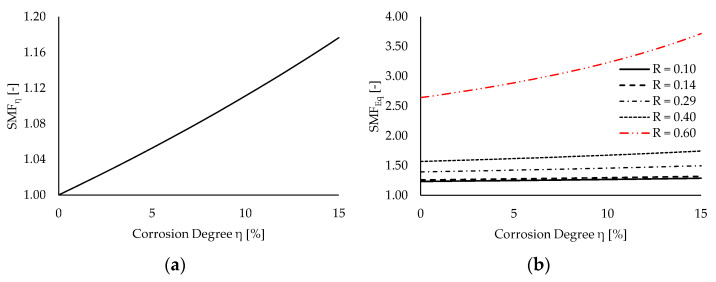
Trends of SMFs for increasing values of corrosion degree *η*: (**a**) corrosion-depending *SMF_η_* and (**b**) mean-stress-depending *SMF_Eq_*.

Figure 7 depicts the values of *SMF_η_* (Figure 7a) and *SMF_Eq_* (Figure 7b) for the increasing corrosion degrees and different values of stress ratio *R*. For the sake of clarity, a nominal stress range Δ*σ*_0_ = 160 N/mm^2^ (i.e., the stress range associated with the *S3* experimental test) is considered to represent values of *SMF_Eq_* against *η*.

One can notice that the *SMF_η_* dependence on the corrosion degree is almost linear for moderate corrosion levels (i.e., *η* ≤ 15%). This outcome depends on the range of investigated values of *η*, as the following approximation holds for the corrosion degree tending to zero (Equation (21), i.e., the McLaurin expansion of Equation (19)):

(21) $$SMF_{\eta }\left(t\right)=\frac{1}{1-\eta_{A}\left(t\right)}\;\approx \;1+\eta_{A}\left(t\right)\;\mathrm{for}\;\eta_{A}\;\to \;0$$

Conversely, a quick increase of SMF_η_ is predicted for higher values of *η*, as Equation (19) clearly displays a hyperbolic trend. For instance, halving the resisting cross-section results in *SMF_η_* = 2, while *η* = 66% is sufficient to triplicate local stresses.

On the other hand, the *SMF_Eq_* trend against *η* significantly varies in dependence on the stress ratio *R*. Specifically, for *R* < 0.40, the corrosion dependence is again basically linear, while for higher values of *R* corrosion the sensitivity becomes more than linear. Specifically, for *R* = 0.1, an increase in *SMF_Eq_* of ≈4% is attained for *η* = 15%, while the same corrosion degree causes *SMF_Eq_* to increase by ≈41% with respect to pristine conditions for *R* = 0.6.

This outcome suggests that the interaction between the corrosion and the mean stress effects is enhanced in the case of significant tensile mean stresses, resulting in a highly detrimental effect on the fatigue performance of steel components in aggressive environments. Indeed, this condition is typical for structural details adopted in steel bridges, for which common stress ratios range within 0.1 ÷ 0.5 due to the relevant permanent loads [16,37].

**Figure 8:**
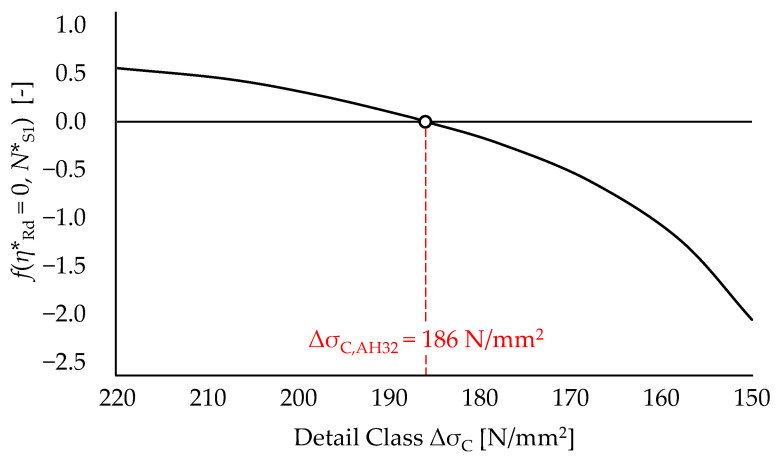
Trend of *f*(η*_Rd_ = 0, N*_S__1_) for decreasing values of Δ*σ_C,AH32_* (Specimen *S1*).

In light of Equations (16) and (19), the “equivalent” load spectrums for each specimen are hence completely defined. Prior to estimating η*_Rd_ for each specimen by solving Equation (8), an “equivalent” detail class Δ*σ_C,AH32_* is preliminarily derived with reference to the pristine AH32 coupon (Specimen *S1*). Owing to the absence of geometrical stress raisers in the case of round coupons, according to EN1993:1–9 [3], inverse logarithmic slopes *m*_1,0_ = *m*_2,0_ = 5 are selected for the S-N curve. Therefore, the proper value of Δ*σ_C,AH32_* is evaluated by finding root of Equation (8) under the assumptions of (i) null corrosion and (ii) number of cycles up to failure N*_S__1_ = 11,500 (see Table 2). The values of *f*(η*_Rd_ = 0, N*_S__1_) are depicted in Figure 8 against the decreasing values of the “equivalent” detail class.

One can notice that iterative solution of Equation (8) for null corrosion yields an “equivalent” detail class for AH32 pristine coupons Δ*σ_C,AH32_* = 186 N/mm^2^. Notably, this value is only slightly higher (+3.3%) than the detail class provided by [3] for plain steel members subjected to normal stresses (i.e., Δ*σ_C_* = 180 N/mm^2^). Hence, the related values of VLCF limit and CAFL are equal to Δ*σ*_10000,*AH32*_ = 506 N/mm^2^ and Δ*σ_D,AH32_* = 155 N/mm^2^, respectively.

**Figure 9:**
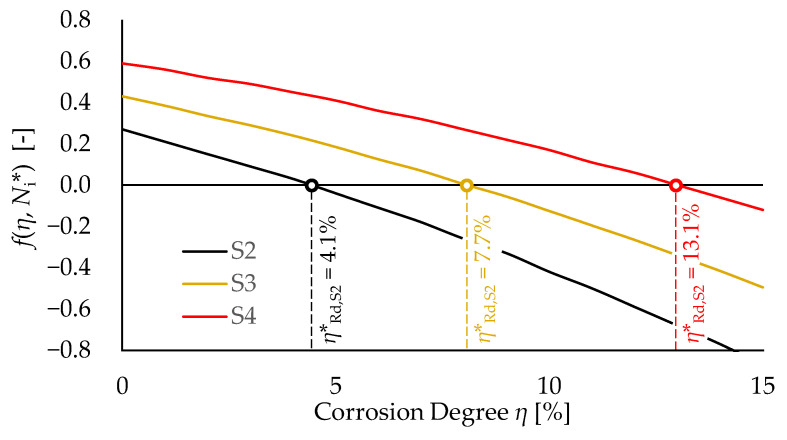
Trend of *f*(η*_Rd_, N*_Si_) for increasing values of maximum corrosion degree for each considered corroded specimen (*S2*-to-*S4*).

With regard to the corroded specimens *S2*-to-*S4*, the trends of *f*(*η*, N_i_*) and the estimated critical corrosion degrees for each test are summarized in Figure 9 and Table 3, respectively. The values of η*_Rd_ are also compared with corresponding the values of *η*_max_ reported in [24].

One can observe that the predicted values of η*_Rd_ are in good agreement with the maximum degrees of artificially induced corrosion for specimens *S2*-to-*S4*. Indeed, the resulting errors are always smaller than 10%. Compliance between the measured and the predicted values of corrosion provides a first validation of the presented procedure with respect to single components subjected to corrosion HCF+LCF fatigue.

The highest error is associated with specimen *S4* (+9.3%), for which the critical corrosion degree is slightly overestimated. This outcome probably depends on the assumption of uniform corrosion, which becomes less accurate for increasing levels of material loss.

Indeed, as corrosion was artificially induced by means of direct current supply, thus higher corrosion degrees correspond to higher exposition times in an aggressive environment. As a result, moderate pits become more likely even for small gauge lengths, owing to the extreme sensitivity of corrosion to evolving boundary conditions.

Therefore, Equations (19) and (20) lead to an underestimation of SMFs for specimen *S4*, resulting in a higher critical corrosion degree with respect to the test conditions.

Table 3 also reports the values of the damage functions *f* for *η* = 0 (henceforth also referred to as “*f*_0_*”*), i.e., the vertical intercepts of *f* for specimens *S2*-to-*S4*, which represent the amounts of corrosion-depending damage. Indeed, each specimen *SX* would present a residual fatigue capacity equal to *f*_0,*SX*_ in the absence of material degradation.

One can notice that, consistently, lower values of *f*_0_ are associated with higher stress ranges. Moreover, higher values of *f*_0_ correspond to higher levels of critical corrosion.

It is worth remarking that the latter condition does not occur in general but, rather, depends on the exposition conditions. On principle, for the same element subjected to different stress histories, corrosion fatigue failure can occur for lower values of η*_Rd_ even in the case of lower stress ranges, if the exposure conditions are significantly worse (e.g., passing from C3 to C5). Therefore, the selection of the appropriate corrosivity category when dealing with a full-scale steel structure in an aggressive environment is a key aspect as well.

**Figure 10:**
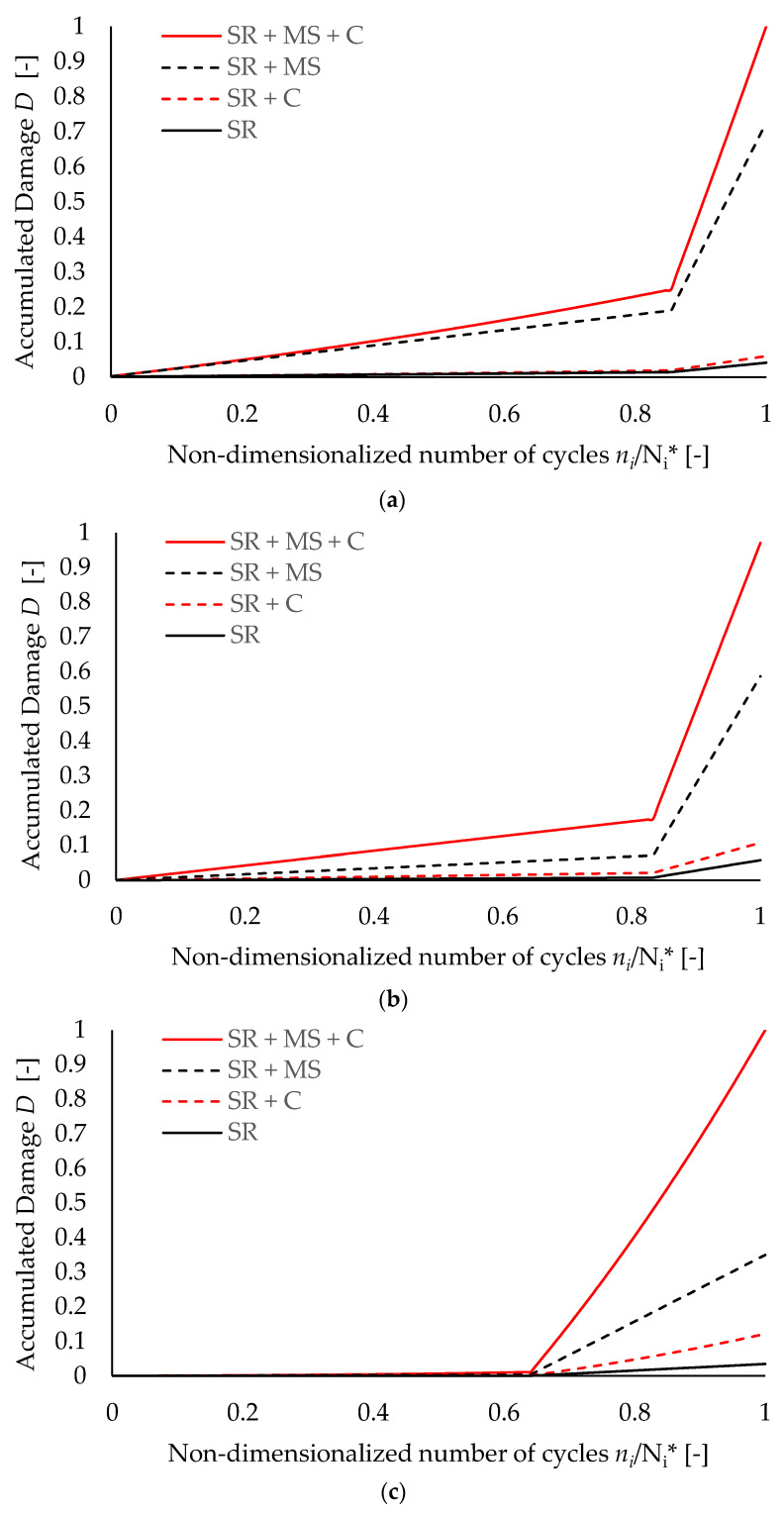
Damage evolution for each test derived by considering/neglecting corrosion and/or mean stress effects: (**a**) specimen *S2* (η*_Rd_ = 4.1%), (**b**) specimen *S3* (η*_Rd_ = 7.7%), and (**c**) specimen *S4* (η*_Rd_ = 13.1%).

As the proposed methodological approach has been applied with respect to post-failure analyses, fatigue checks in the form “*η_Ed_*(*t*) ≤ *η_Rd_*(*t*)” do not have a clear significance. Nevertheless, it is still worth highlighting the influence of each damage source (i.e., cyclic loading, mean tensile stress, and corrosion) on the overall corrosion fatigue performance of each specimen. Figure 10 depicts the damage evolution for specimens *S2*-to-*S4* against the increasing values of the endured cycles *n*_i_ up to failure (normalized with respect to *N*_i_*).

Damage sources are discerned by considering four scenarios for each test, namely:

(i). stress range + mean stress effect + corrosion (total damage, SR + MS + C);
(ii). stress range + mean stress effect (damage in pristine conditions, SR + MS);
(iii). cyclic loadings + corrosion (corrosion damage under alternating loads, SR + C);
(iv). stress ranges with no corrections (damage according to EN1993:1–9, SR).

For the sake of clarity, damage scenarios in which corrosion is accounted for are always related to a maximum corrosion degree equal to η*_Rd_.

One can observe that, for all specimens, damage contributions which can be ascribed to corrosion and mean stress effects do not simply add up but, rather, amplify each other, leading to premature failure. Indeed, for a given number of cycles, the sum of the damage aliquots for the SR + MS and SR + C scenarios is always smaller than the corresponding damage in the SR + MS + C scenario.

Nevertheless, the maximum corrosion-related damage (SR + C scenarios) coherently increases for specimens having higher values of η*_Rd_, while the corresponding mean-stress-related damage (SR + MS scenarios) decreases as expected.

Moreover, the knee points in the damage evolution curves can be clearly noticed at the transition from the HCF to the LCF test conditions, highlighting how plastic deformations rapidly reduce the residual fatigue life of steel components. This condition is mostly evident for specimen *S4* (see Figure 9c), for which damage under purely elastic stresses is almost null, even in an aggressive environment (*D* ≈ 0.01 at the knee point for the SR + MS + C scenario). This outcome clearly depends on the applied HCF stress range (Δ*σ_0,_*_1,S4_ = 100 N/mm^2^), which is the lowest among all the specimens, while the applied mean stress is unchanged.

Finally, it is worth highlighting how the evaluation of damage with no correction on stress ranges (SR scenarios) leads to severe overestimations of the residual fatigue life. Indeed, the highest value of “unaltered” damage, which is shown by specimen *S3* (*D* ≈ 0.06 for *n_i_*/N_i_* = 1, see Figure 10b, black solid line), is still basically negligible, suggesting that the damage calculations performed according to the EN1993:1–9 [3] prescriptions may be highly non-conservative when dealing with existing structures affected by significant material degradation.

## 4. Parametrical Analyses

In order to investigate the influence of the test conditions and material properties on the predicted values of the critical corrosion degree, parametrical analyses are hence performed based on the results for specimen *S4*. The most corroded coupon is selected to yield more noticeable differences in terms of η*_Rd_ when the model parameters are changed.

In particular, the present parametrical study is performed by varying the following quantities: (i) stress ratio of the HCF block *R*_1_, (ii) stress ratio of the LCF block *R*_2_, (iii) AH32 tensile strength *f_u_*, and (iv) assumed corrosivity category C*X*. The selected ranges of variation for the parametrical analyses are summarized in Table 4.

For the sake of clarity, the influence of each single parameter is investigated assuming the other quantities are in compliance with the test conditions from [24].

**Figure 11:**
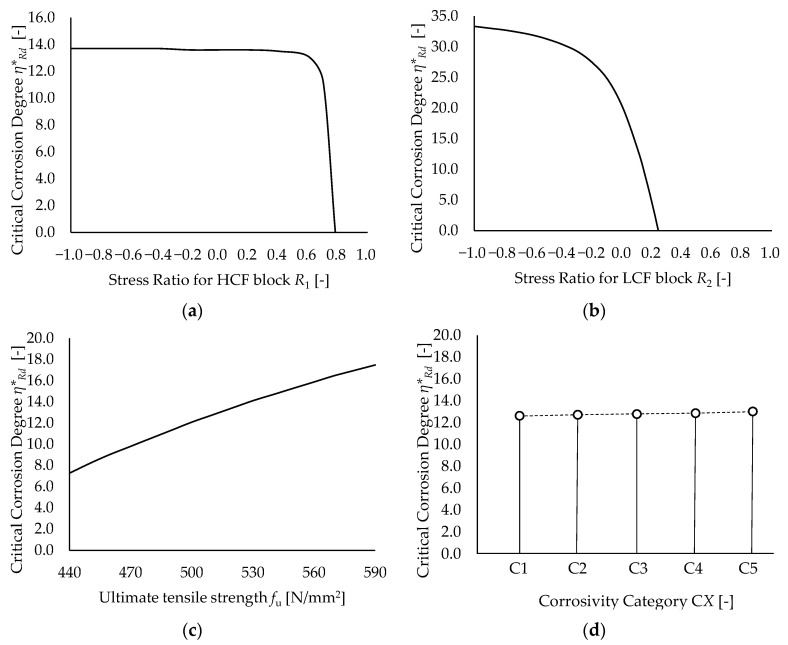
Results of parametrical analyses based on specimen *S4* in terms of estimated η*_Rd_: (**a**) influence of stress ratio of the HCF block *R*_1_, (**b**) influence of stress ratio of the LCF block *R*_2_, (**c**) influence of AH32 tensile strength *f_u_*, and (**d**) influence of corrosivity category C*X*.

The results of the parametrical analyses in terms of the estimated critical corrosion degrees for increasing values of the varied quantities are depicted in Figure 11.

One can notice that the influence of HCF block stress ratio *R*_1_ on the predicted values of the critical corrosion degree is almost negligible (see Figure 11a). Indeed, for −1 ≤ *R*_1_ ≤ 0.6, the estimated η*_Rd_ is basically constant (i.e., between 13 ÷ 14%) before quickly dropping to zero for *R*_1_ = 0.78. Such a threshold value of *R*_1_, for which fatigue failure is predicted in the absence of material degradation, is non-representative of the actual HCF performance of specimen *S4* as it is close to the asymptotic condition of static loading (*R*_1_ = 1).

Notably, this outcome is consistent with the results reported in Section 3. Indeed, specimen *S4* exhibited insignificant damage due to corrosion HCF (*D* ≈ 0.01 at the knee point, see Figure 10c, SR + MS + C scenario).

Conversely, LCF block stress ratio *R*_2_ has a significant influence on the predicted values of η*_Rd_ (see Figure 11b) Namely, in the case of reversed corrosion LCF (i.e., for *R*_2_ = −1), the resulting critical corrosion degree (33.3%) is ≈2.5 times higher with respect to the reference value (η*_Rd_ = 13.1% for *R*_2_ = 0.1). On the other hand, the critical corrosion degree rapidly drops to zero for R_2_ = 0.26. This clearly highlights how the fatigue performance of specimen *S4* is governed by the simultaneous effect of corrosion, local plasticization, and tensile mean stress.

With regard to UTS, an increase of *f_u_* within the range of plausible values for AH32 steel (i.e., 440 ÷ 590 N/mm^2^) results in a corresponding increase of the critical corrosion degree, as expected (see Figure 11c). Indeed, as shown by Equation (18), the higher values of *f_u_* contribute to mitigating the effect of the mean stresses (both the loads- and the corrosion-related ones).

Nevertheless, the sensitivity of η*_Rd_ to UTS variations is limited with respect to the influence of *R*_2_. Specifically, for *f*_u_ = 440 ÷ 590 N/mm^2^, the predicted values of η*_Rd_ are in the range 7.3 ÷ 17.5%. This outcome depends on (i) the less-than-linear dependence of 1/*SMF_Eq_* from *f_u_* (Equation (20)) and (ii) the relatively small scatter associated with UTS for structural steels. Conversely, higher dispersions are associated with stress histories due to the intrinsic randomness of transient loads (e.g., traffic loads, wind actions, and earthquakes [16,37]).

Finally, no significant variations can be appreciated in terms of η*_Rd_ owing to a change of the corrosivity category *CX* (see Figure 11d). Indeed, the estimated critical corrosion degrees always fall in the range of 12.6 ÷ 13.1% for each considered category. This result descends from the peculiar damage evolution shown by specimen *S4*. Indeed, as reported in Section 3, corrosion fatigue damage is basically condensed in the lone LCF part. Therefore, the influence of the corrosive process becomes relevant only when higher values of *η* have been already attained. At this point (i.e., for *t* >> 0), for a given value of η*_Rd_, Equation (1) yields similar values of *η*(*t*) for all corrosivity categories.

Nevertheless, it should be remarked that this outcome should not be considered true in general as steel structures in aggressive environments can be subjected to significant loads even shortly after erection time. In that case, low levels of corrosion can also detrimentally affect the cyclic performance of the steel members, resulting in a more pronounced sensitivity with respect to the corrosivity category of concern [29,36].

## 5. Conclusions

A simplified methodology for the corrosion fatigue assessment of steel structures in aggressive environments was presented in this work. The proposed method was hence first validated against corrosion HCF+LCF tests drawn from the literature [24]. In light of the results, the following conclusions can be drawn:

- The proposed method for the corrosion fatigue analysis of steel structures is based on the concept of “critical” corrosion degree η*_Rd_, i.e., the minimum corrosion value which induces corrosion fatigue failure for a selected target fatigue life t*;
- The critical corrosion degree allows expressing fatigue verification in a “demand vs. capacity” form in line with the aims of performance-based engineering;
- Corrosion HCF+LCF tests carried out by Sun et al. [24] were selected to provide a first validation of the presented methodology;
- The estimated values of η*_Rd_ are in good agreement with the test conditions. The prediction errors are always lower than 10%, despite the considered simplifications (+9.3% in the case of specimen *S4*);
- Parametrical analyses were performed starting from the results for the most corroded specimen (*η*_max_ = 12%). The estimated values of η*_Rd_ (0.0 ÷ 33.3%) showed a significant sensitivity with respect to LCF stress ratio *R_2_*;
- UTS also significantly affects the corrosion fatigue performance of specimens as higher values of *f_u_* mitigate the detrimental effect of mean stress (η*_Rd_ = 7.3 ÷ 17.5% for *f_u_* = 590 ÷ 440 N/mm^2^);
- The critical corrosion degree exhibited no significant sensitivity with respect to both HCF block stress ratio *R*_1_ and corrosivity category C*X*, although this result probably depends on the particular test conditions assumed in [24];
- The reliability of the presented methodology will be further proved with regard to full-scale steel structures in aggressive environments.

## Figures and Tables

**Table 1:** Suggested values of C for each corrosivity category according to [27].

Corrosivity Category	Exposition	C [-]
C1	Very low	5
C2	Low	3.5
C3	Medium	2.5
C4	High	1.5
C5	Very high	1

**Table 2:** Test conditions for corrosion HCF+LCF tests on AH32 steel performed by Sun et al. [24] (Specimens *S1–S4*).

Specimen	*S1*	*S2*	*S3*	*S4*
Gauge Length *L_g_* [mm]	30	30	30	30
Gauge Diameter *D_g_* [mm]	6	6	6	6
Max. Corrosion Degree *η*_max_ [%]	0	4	8	12
Number of Cycles (First Block) *n*_1_ [-]	10,000	10,000	10,000	10,000
Nominal Stress Range (First Block) Δ*σ*_0,__1_ [N/mm^2^]	300	220	160	100
Nominal Mean Stress (First Block) *σ_m,_*_1_ [N/mm^2^]	200	200	200	200
Stress Ratio (First Block) *R*_1_ [-]	0.14	0.29	0.40	0.60
Number of Cycles (Second Block) *n*_2_ [-]	≈1500	≈1700	≈2000	≈5600
Nominal Stress Range (Second Block) Δ*σ_0,_*_2_ [N/mm^2^]	450	400	360	330
Nominal Mean Stress (Second Block) *σ_m,_*_2_ [N/mm^2^]	247.5	220	198	181.5
Stress Ratio (Second Block) *R*_2_ [-]	0.10	0.10	0.10	0.10

**Table 3:** Estimated values of critical corrosion degree for specimens *S2*-to-*S4*.

Specimen	*S2*	*S3*	*S4*
N_i_*	11,700	12,500	15,600
*η*_max_ [24]	4	8	12
η*_Rd_	4.1	7.7	13.1
Percentage Error [%]	+2.1%	−3.9%	+9.3
*f*(*η* = 0; N_i_*) [-]	0.27	0.43	0.59

**Table 4:** Assumed ranges of variation for *R*_1_, *R*_2_, *f_u_*, and C*X* for parametrical analyses.

Parameter	Reference Value [22]	Range of Variation
*R*_1_ [-]	0.60	−1 ÷ 1
*R*_2_ [-]	0.10	−1 ÷ 1
*f_u_* [N/mm^2^]	515	440 ÷ 590
*CX* [-]	C5	C1 ÷ C5

## Data Availability

The data used in this study to support presented findings are included within the article.
